# Understanding current UK practice for the incidental identification of vertebral fragility fractures from CT scans: an expert elicitation study

**DOI:** 10.1007/s40520-022-02124-w

**Published:** 2022-04-18

**Authors:** Garima Dalal, Paul A. Bromiley, Eleni P. Kariki, Shawn Luetchens, Timothy F. Cootes, Katherine Payne

**Affiliations:** 1 Manchester Centre for Health Economics, University of Manchester, Oxford Road, Manchester, UK; 2 Centre for Imaging Sciences, University of Manchester, Manchester, UK; 3 Manchester University NHS Foundation Trust, Manchester, UK; 4 Optasia Medical Limited, Manchester, UK

**Keywords:** Incidental identification, Expert elicitation, Vertebral fragility fractures, Osteoporosis, Artificial intelligence, Diagnosis, Prevention, CT, Radiological

## Abstract

**Background:**

There is an emerging interest in using automated approaches to enable the incidental identification of vertebral fragility fractures (VFFs) on existing medical images visualising the spine.

**Aim:**

To quantify values, and the degree of uncertainty associated with them, for the incidental identification of VFFs from computed tomography (CT) scans in current practice.

**Methods:**

An expert elicitation exercise was conducted to generate point estimates and measures of uncertainty for four values representing the probability of: VFF being correctly reported by the radiologist; the absence of VFF being correctly assessed by the radiologist; being referred for management when a VFF is identified; having a dual-energy X-ray absorptiometry (DXA) scan after general practitioner (GP) referral. Data from a sample of seven experts in the diagnosis and management of people with VFFs were pooled using mathematical aggregation.

**Results:**

The estimated mean values for each probability parameter were: VFF being correctly reported by the radiologist = 0.25 (standard deviation (SD): 0.21); absence of VFF being correctly assessed by the radiologist = 0.89 (0.10); being referred for management when a VFF is identified by the radiologist = 0.15 (0.12); having a DXA scan after GP referral = 0.66 (0.28).

**Discussion:**

These estimates could be used to facilitate the subsequent early economic evaluation of potential new approaches to improve the health outcomes of people with VFFs.

**Conclusion:**

In the absence of epidemiological studies, this study produced point estimates and measures of uncertainty for key parameters needed to describe current pathways for the incidental diagnosis of VFFs.

**Supplementary Information:**

The online version contains supplementary material available at 10.1007/s40520-022-02124-w.

## Introduction

The economic burden of fragility fractures was estimated to be €37.5 billion across Spain, Germany, France, UK, Italy and Sweden in 2017 [1]. In the UK, osteoporosis is projected to cost over £5.5 billion by 2025 [2]. Among the different osteoporotic fragility fractures, vertebral, forearm and hip fragility fractures are the most common [2, 3]. Vertebral fragility fractures (VFFs) are known to be associated with a loss in health-related quality of life as a result of pain, deformity, loss of independence and early death [4]. They are also known to be an early indicator of increased risk of hip fractures [5–7], which are associated with significant morbidity and mortality [8]. Evidence suggests that prevalent VFFs increase the risk of a subsequent hip fracture by twofold and the risk of a subsequent VFF by fivefold in the first year [5, 6, 9].

Consequently, the accurate and timely identification of VFFs is required to enable starting an effective bone health management strategy involving, for example, bisphosphonates (among other treatment alternatives available, such as denosumab, PTH 1–34 [10]) supported by dietary advice, vitamin D supplementation and exercise fall prevention programmes [11, 12]. Current practice relies on radiologists identifying VFFs on medical images and there is evidence to suggest that up to 70% of VFFs are missed by radiologists when looking at medical imaging obtained for other clinical indications [13, 14]. The reasons for these missed diagnoses of VFFs by radiologists or primary care physicians are not known but could be a number of factors, such as incidental findings from medical imaging often not being reported, a lack of standardization in the definition of VFFs (various clinical and morphometric methods exist to confirm their presence), and the clinically “silent” nature of VFFs, as they often do not cause symptoms that are suggestive of a fracture or the symptoms are assumed to be common in the patient population affected by VFFs.

Within this context, there is emerging interest in using automated approaches to enable the incidental identification of VFFs on existing medical images such as computed tomography (CT) scans visualising the spine [15]. The widespread availability of CT scans, offering high image resolution, provides an opportunity to develop an automated approach for the incidental identification of VFFs using machine learning (ML)-based computer-aided diagnostic (CAD) systems. The ultimate goal of such approaches is to accurately identify and grade the severity of VFFs to direct older people at risk of future fragility fractures onto timely bone health management programmes via their general practitioner (GP) or Fracture Liaison Service (FLS).

Recently, a national standards framework for Digital Health Technologies has emerged in NHS England [16]. This framework requires evidence of clinical and cost-effectiveness as a pre-requisite to enable the implementation of ML-CAD systems, such as the ones to identify VFFs, into clinical practice [16]. Evidence of clinical effectiveness can come from numerous sources including randomised trial data and observational data from cohorts of patients [17]. Decision-analytic model-based cost-effectiveness analysis (CEA) is a useful tool in the development phases of new diagnostic or treatment options [18, 19]. Early CEA provides technology developers with evidence on how to best place the new healthcare intervention in current pathways of care and understand what additional evidence is needed to maximise potential benefits to the patient population [18].

Using early CEA provides a structured framework to use data from multiple sources to understand the potential costs to healthcare systems and benefits to patient populations of introducing a new healthcare intervention into practice [20]. When CEA is conducted very early in the development phase of a new healthcare intervention, data available from randomised trials or observational studies will be limited and often not available. This absence of data is particularly likely for interventions, such as new medical and diagnostic devices [19], and has stimulated the reliance on the use of expert judgement [21] which first emerged in applications in ecology, environment and engineering [22, 23] and now has application in the healthcare context [24, 25].

To move towards an early CEA of emerging digital healthcare interventions, such as ML-CAD systems to diagnose VFFs, it is crucial to be able to clearly describe current practice using the best evidence available, which may sometimes be expert judgement. There are some available data that quantify VFF incidental identification rates by radiologists when reading CT scans and the current practice care pathway for patients with a VFF. However, patient populations in these studies differ. This hampers early attempts to understand the potential cost-effectiveness of ML-CAD systems meaning that it is challenging to move towards implementing such approaches into healthcare systems. In this context, this study aimed to use a structured expert elicitation exercise to generate point estimates and measures of uncertainty for key values needed to understand the potential healthcare costs and health consequences of ML-CAD systems to identify VFFs from CT scans in NHS England. From here on, the identification of VFFs refers to the incidental identification of VFFs on CT imaging examinations not specifically performed to review the spine.

## Methods

A structured expert elicitation exercise was conducted following best practice guidelines [26, 27] and reported using a published checklist (see reporting criteria in Online Resource 1) [28] to generate estimates of pre-specified values needed to conduct a subsequent CEA of a ML-CAD system to identify VFFs from CT scans that have been performed for a clinical indication not related to the spine (but where the spine is visible) in NHS England. The process of conducting a structured expert elicitation exercise involves asking an individual defined as an ‘expert’ to provide a range of estimates, based on their experiences, for a given unknown value such as an input parameter for a CEA (hereafter ‘parameter’). The estimates gathered from each expert are first used by the analyst to calculate a mean value and the variation associated with it in the form of a probability distribution. These calculated estimates are then aggregated (or ‘pooled’) across experts to provide an overall mean value and the uncertainty associated with it for each parameter.

Ethical approval was not required for this study according to the University Ethics Decision Tool as personal identifiable or sensitive information was not collected and participants were not considered to be vulnerable or at risk of disclosing illegal or unprofessional conduct. Data generated from the interviews were anonymised after completing the exercise and no financial incentive was offered to the experts.

### Elicited parameters

**Fig. 1:**
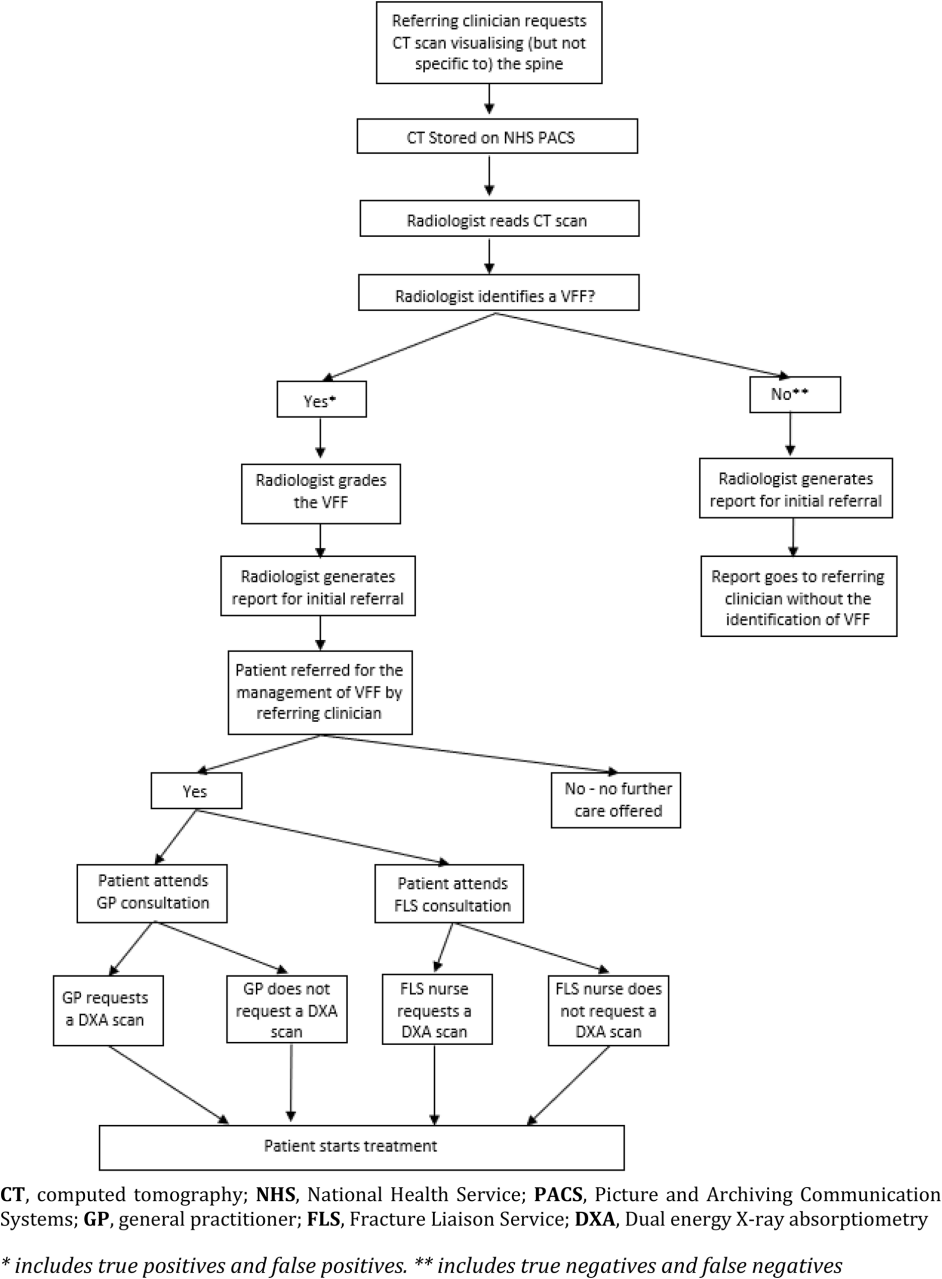
Standard care pathway for vertebral fragility fracture (VFF) identification in current practice

Four pre-specified parameters for this study that required estimation were identified (see Online Resource 2 and Table 1) by conceptualising a standard pathway of care for a patient living in England from the point of VFF identification to treatment. This pathway of care (see Fig. 1) was conceptualised with input from a NHS radiologist and two academic computer scientists with an interest in the use of AI in radiology.

A literature search confirmed the absence of relevant published evidence for the four pre-specified values. This review identified a lack of published consensus regarding the true proportion of undiagnosed VFFs. Some empirical evidence exists that suggests only around one-third of osteoporotic VFFs visualised in medical imaging come to clinical attention; however, a wide variation exists with some studies suggesting an identification rate of less than 15% [14, 29–32]. Low referral rates for the management of VFFs have also been documented. A recent multi-site study found that the median FLS referral rate across four UK hospitals was only 13% [29]. Similarly, Howlett and colleagues reported that recommendations for further referral/management were made in only 2.6% of the patients with a VFF in an audit covering 127 radiology departments across the UK [33]. This suggests that referral rates in hospital are low regardless of whether the patients are referred to their GP or FLS. There was no published evidence that reported the likelihood of having a dual-energy X-ray absorptiometry (DXA) scan upon referral to a GP.

### Expert identification and selection

For the purpose of this study, an expert was defined as a healthcare professional involved in the identification or management of VFFs and/or osteoporosis or academic researchers involved in producing evidence to inform the diagnosis and management of VFFs and/or the development of new ML-CAD systems. The experts were chosen on the basis of their knowledge of the topic and interest. There is no published guidance on the required number of experts to take part in an elicitation exercise. However, there is general guidance that the type of expert is just as important as the number of experts [26, 27]. Published studies using expert elicitation have used sample sizes of two to three experts [34–36]. Our focus was to ensure we identified experts with the relevant background and knowledge on the topic of interest with a minimum sample size of three experts per parameter. Eight experts were identified through existing links and collaborations within the research team as well as publicly available websites. Seven of these experts were also involved in academic research. It was deemed important to ensure a range of disciplines were represented in the expert group.

### Elicitation method

The pre-specified protocol (Online Resource 2) was used to guide the elicitation exercise which followed good practice recommendations [26]. Data on the unknown parameters were collected from individual experts using the ‘quartile method’ that asks each expert to specify the highest and lowest plausible values (i.e. a plausible range), a median and upper and lower quartile values (see Online Resource 4). The quartile method is suitable for eliciting parameters that are proportions and encourages experts to think and minimises anchoring effects [27, 37]. It is also recommended by expert elicitation guidelines [27]. The elicitation exercise was piloted using the first expert. No changes were made to the elicitation exercise after the pilot.

### Data collection

All experts who were willing to take part were invited via email to participate in face-to-face or semi-structured telephone interviews on an individual basis with one researcher (GD). The interviewer guided the expert through the elicitation exercise using a set of closed and open-ended questions to elicit the point estimates and distribution of the four parameters. The elicitation exercise sheet (see Online Resource 4) was used to facilitate the exercise by showing it to the expert during face-to-face interviews. Experts who participated via a telephone interview were asked to have the elicitation sheet open in front of them on a computer screen. Responses were not shared between participants and no references were made to published values to prevent influencing experts’ judgement.

Prior to the interview, a copy of the elicitation exercise sheet and details about the project were shared with the experts. The elicitation exercise involved first explaining the task and the relevant terminology to the expert, followed by an example exercise unrelated to the research problem to aid understanding of the exercise. Experts were then asked to consider a population of individuals aged 70 years who have been referred for a CT scan in the NHS and provide values for the specified parameters. The questions were asked in the format of, for example, “imagine a cohort of 100 patients with a VFF, how many do you think would have their VFFs correctly reported by the radiologist?” Due to time constraints, it was not feasible to elicit all four parameters from all experts. Hence, two to four parameters were pre-selected for each expert to reduce the cognitive burden.

### Data aggregation

Data from each expert were summarised and data from the total sample of experts were pooled using mathematical aggregation. Following standard practice [21, 28, 38], a beta distribution was fitted to the values elicited from each expert for each parameter using the SHELF 2.0 package [27] run using the statistical software RStudio [39]. The distributions were mathematically aggregated using equal weights for each expert for all parameters. The statistical software WinBUGS [40] was used to linearly pool the experts’ beliefs using an unweighted average of their probability distributions. Linear pooling was considered to be appropriate rather than using the alternative, logarithmic opinion pooling, that is known to produce narrower distributions. Specifying a narrower distribution around the point estimate for each parameter implies each expert is more certain and logarithmic pooling may suggest perhaps unrealistic consistency across the sample of experts [26, 41].

The WinBUGS model was run using two Monte Carlo Markov chains with an initial ‘burn-in’ of 10,000 iterations which was determined using the Gelman–Rubin convergence diagnostic and a visual assessment of trace plots of the sample values. A further 20,000 iterations were run after the ‘burn-in’ based on the accuracy of the posterior aggregated estimates which was assessed by verifying that the estimated Monte Carlo errors of the pooled distributions were less than 5% of the sample standard deviations. The summary measures obtained from the pooled distributions (mean and standard deviation) were used to calculate the alpha and beta values of the beta distribution for each of the four parameters using the method of moments.

## Results

Seven out of eight experts who were contacted agreed to participate in the elicitation exercise. The experts represented different parts of the UK including the North West (*n* = 3), East Midlands (*n* = 1), West Midlands (*n* = 1), South West (*n* = 1) and Scotland (*n* = 1). The disciplines represented in the expert group also varied with experts specialising in endocrinology (*n* = 1), rheumatology (*n* = 1), radiology (*n* = 1), ortho-geriatric medicine (*n* = 1), imaging science (*n* = 1), general practice (*n* = 1) and ML-CAD systems (*n* = 1). Five experts were interviewed by telephone and interviews were conducted face-to-face with two experts. All interviews took place in February 2020. The mean length of time to complete the exercise was 1 h. The values elicited from all seven experts were included in the final analyses as none provided implausible values and all completed the exercise.

### Elicited parameter values

Each of the seven experts provided estimates for 2–4 parameters and a probability distribution was created for each expert for each parameter representing the probability of: VFF being correctly reported by the radiologist; absence of VFF being correctly assessed by the radiologist; being referred for management when a VFF is identified; having a DXA scan after GP referral (see Online Resource 3). These results also show the degree of uncertainty around the point estimates for each parameter. The results indicated that experts were most uncertain about the probability of having a DXA scan after GP referral and the probability of VFF being correctly reported by the radiologist (radiologist sensitivity). There was a high discrepancy between the observed expert values. In contrast, there was consistency between the elicited values for the probability of an absence of VFF being correctly assessed by the radiologist (radiologist specificity). The estimated distribution parameters per expert for each of the four parameters are provided in Table 1.

**Table 1:** Distribution parameters for the elicited quantities

Parameter	Beta distribution parameters^a^						
	Expert ID1	Expert ID2	Expert ID3	Expert ID4	Expert ID5	Expert ID6	Expert ID7
Probability of VFF being correctly reported by the radiologist	*α* = 1.61 *β* = 1.84	Not elicited	*α* = 0.74 *β* = 2.93	Not elicited	*α* = 3.96 *β* = 30.72	*α* = 3.05 *β* = 10.54	Not elicited
Probability of absence of VFF being correctly assessed by the radiologist	*α* = 5.25 *β* = 1.32	Not elicited	*α* = 45.86 *β* = 6.46	Not elicited	Not elicited	*α* = 23.02 *β* = 1.36	*α* = 122.40 *β* = 7.28
Probability of being referred for management when a VFF is identified by the radiologist	*α* = 0.75 *β* = 7.08	*α* = 3.01 *β* = 23.72	Not elicited	*α* = 0.82 *β* = 5.68	*α* = 4.29 *β* = 10.38	Not elicited	*α* = 10.29 *β* = 90.64
Probability of having a DXA scan after GP referral	*α* = 13.27 * β* = 1.54	*α* = 5.40 *β* = 1.52	Not elicited	*α* = 1.38 *β* = 4.58	Not elicited	Not elicited	*α* = 102.20 *β* = 34.23

*VFF* vertebral fragility fracture, *DXA* dual-energy X-ray absorptiometry, *GP* general practitioner

^a^Beta distribution is a continuous probability distribution used to model continuous random variables defined on a scale of 0 to 1. The beta distribution is described by two parameters, alpha (*α*) and beta (*β*), which govern the shape of the distribution

### Data aggregation

Table 2 reports final estimates with measures of variation for each of the four parameters for the total sample of experts. These values were also used to fit summary distributions to represent the aggregated elicited values for each parameter (see Online Resource 4).

**Table 2:** Estimates and distribution for each of the four parameters

Parameter	Mean	Standard deviation	2.5th percentile^a^	Median estimate	97.5th percentile^a^
Probability of VFF being correctly reported by the radiologist	0.253	0.209	0.014	0.183	0.800
Probability of absence of VFF being correctly assessed by the radiologist	0.891	0.101	0.588	0.922	0.992
Probability of being referred for management when a VFF is identified by the radiologist	0.146	0.118	0.004	0.111	0.451
Probability of having a DXA scan after GP referral	0.663	0.281	0.049	0.756	0.980

*VFF* vertebral fragility fracture, *DXA* dual-energy X-ray absorptiometry, *GP* general practitioner

^a^2.5th and 97.5th percentiles are the values below which 2.5% and 97.5% of the observations may be found, respectively. 95% of the values lie within the 2.5th and 97.5th percentiles

On average, the experts indicated that the mean value of the probability of the VFF being correctly reported by the radiologist was 25% suggesting that a very low proportion of VFFs are incidentally identified. In contrast, the probability of an absence of VFF being correctly assessed by the radiologist was believed to be 89%. These estimates are intuitive because it is perhaps easier to establish the absence of VFFs compared with identifying visible VFFs and determining whether they would be classed as VFFs according to various techniques. The estimated probability of referral after being diagnosed with a VFF by the radiologist was only 15%, meaning that a significant proportion of VFFs would likely remain untreated even when identified on CT scans in current practice. The probability of a having a DXA scan after GP referral was considered to be significantly low which suggests that some patients seeing a GP may be starting treatment without a baseline bone mineral density (BMD). When a VFF is present, a baseline DXA BMD is not compulsory to establish the diagnosis of osteoporosis [3]. Nevertheless, baseline DXA BMD measurements may be required and are often preferred to help assess response to treatment and direct future patient management [42]. In some jurisdictions, vertebral fracture assessment (VFA) can also be used as an alternative to DXA BMD due to its low-cost nature. Clinical guidelines in the UK recommend VFA to establish the presence of any prior VFFs following unexplained height loss of ≥ 4 cm, kyphosis, recent or current long-term use of glucocorticoids or a BMD *T*-score ≤  − 2.5 in postmenopausal women and older men [3].

## Discussion

This study used a structured expert elicitation exercise to generate estimates for key parameters needed to understand current pathways of care to diagnose VFFs. The study showed that it was feasible for experts to provide estimates for parameters, and their associated uncertainty, for which there is a paucity of available data. There was some evidence of parameters published in the literature. However, these studies reported data that did not provide a measure of the uncertainty around the estimates which is required for use in CEAs.

The experts that we consulted indicated that one-fifth of people with a VFF had their VFF correctly reported by radiologists when reading a CT scan depicting the spine. This falls within the wide range that has been reported in the literature which varies from 13 to 34% [29, 31–33, 43]. Additionally, the experts expressed the presence of considerable uncertainty associated with this value (as indicated by the standard deviation) which reflects the wide variation reported to date for this parameter. This discrepancy in the estimates could be due to a number of reasons including differences in the patient population, the specialty of the radiologist reviewing the images [44], the method used for assessing the presence of a fracture, subjectivity in identifying mild fractures, exclusion of mild fractures (some studies only included grade 2 and grade 3 fractures) and a lack of awareness of the need to search for VFFs.

Our finding of 15% of patients with VFFs being referred for management to their GP or FLS is higher than the referral rate reported in the literature [29–33]. This could be due to a number of reasons. Data published by Bromiley et al. only included centres with access to the FLS (thereby did not include GP referrals) [29] and another study also excluded GP referrals with the referrals being restricted to FLS or a clinical service for osteoporosis [33]. The low probability of being referred for management seems to vary and depends on different factors. It may be explained by the perceived lack of importance of VFFs and osteoporosis, particularly when compared to other diseases. For example, in practice, radiologists most often do not include a referral recommendation in their report when a VFF is identified and similarly, referring clinicians tend to mainly focus on the primary reason for the CT scan and thereby disregard the fracture. This is despite the importance of VFFs in the diagnosis of both primary and secondary osteoporosis [3, 45] and, in monitoring the side effects of therapies that may induce secondary osteoporosis [45].

As expected, the probability of having a DXA scan was believed to be low for GP referral (66%), potentially due to the absence of focus on secondary fracture prevention by GP practices. The estimated probability of an absence of VFF being correctly assessed by the radiologist was 89%. The degree of uncertainty, as indicated by the large standard deviation, observed in our study for the probability of being referred when a VFF is identified by the radiologist and the probability of having a DXA scan after GP referral could be due to the experts providing responses based on their own experience in different regions of the country.

There is emerging interest in improving the diagnosis of VFFs and various ML-CAD systems are currently in development. AI-based algorithms to identify VFFs on CT scans have previously been shown to be accurate and sensitive to identifying fractures [15, 46]. These interventions access the medical images stored on Picture Archive and Communication Systems (PACS) and use AI to either identify and/or grade VFFs, or provide a measure of the degree of confidence of a VFF being present on CT scans or radiographs. In addition to detecting VFFs, ML-CAD systems can also be incorporated into a service provided to a healthcare site. One example of this is ASPIRE™ (Optasia Medical Ltd., Cheadle Hulme, Greater Manchester, UK; www.optasiamedical.com) which is a tele-radiology service that accesses CT scans from a PACS and creates sagittal reformations depicting the spine. It then refers patients with an identified VFF to their GP or a local FLS (if an FLS exists in their region). This ensures that patients with diagnosed VFFs are followed up for fracture management and start prompt treatment. Implementing an AI-based system to detect VFFs into practice requires the development of a complex intervention that consists of several interconnected components that present a unique set of problems to researchers evaluating their potential cost-effectiveness [31, 47]. Such complex AI-based interventions pose some specific challenges to ensure the evidence to inform their introduction into clinical practice meets the recommendations published by, for example, the Evidence Standards Framework for Digital Health Interventions [16].

The main strength of this study was that experts from various parts of the UK and with a range of expertise took part in the elicitation exercise. This ensured that the views represented in the pooled distributions were reflective of the potential variation observed across the country and different disciplines. We also assigned equal weight to all experts through linear opinion pooling to combine distributions across experts. This approach to aggregating beliefs ensures that all views are equally reflected in the pooled estimates, and that the pooled parameter distributions are not artificially narrow, which implies a greater decision certainty. Research suggests that different results are obtained depending on the mathematical aggregation approach used [41] and it is unclear which approach performs best. However, linear opinion pooling is more widely used in practice and does not rule out potential parameter values that are deemed implausible by any one expert.

The findings of this study come with some limitations. Currently, there is no set guidance for the required sample size to conduct expert elicitation. Expert selection and sample sizes are often determined based on practicality and representativeness of the experts. Given the small number of individuals with relevant expertise in this field, it was challenging to determine whether the study sample was sufficiently representative. However, it was believed that judgements from key opinions leaders in the field were captured. Sample sizes of less than 10 experts have commonly been reported in elicitation studies with some studies reporting a sample of five to seven experts [21, 48, 49]. A sample size of three experts may appear to be prohibitively small. However, in the field of expert elicitation, a sample size of three may be sufficient provided the individuals have the relevant expertise for the topic of interest. Indeed, published expert elicitation studies have used sample sizes of three or fewer [34–36]. In wider practice when using expert values in model-based CEAs for policy decisions, some studies have relied on a single expert which, although considered bad practice, is often necessary because of time constraints driven by the health technology assessment process [38]. This pragmatic approach to generate estimates of values needed for model-based cost-effectiveness analysis, although often necessary, ignores the uncertainty associated with expert beliefs. Using the structured expert elicitation approach we used in this study, even when conducted with only three experts, provides a means for producing the values necessary in the required format for model-based cost-effectiveness analysis in the absence of data from other sources. There are some recommendations in the literature regarding the number of experts, where three to five experts is considered to be an appropriate number as a larger number of experts may be associated with diminishing marginal returns, meaning that the benefit of an additional expert in the group plateaus beyond a certain threshold [50].

With regard to the conduct of the elicitation exercise, some limitations were inherent due to the practicalities of collecting the data. Telephone interviews meant that it was challenging to ask experts to provide immediate feedback on the distribution of their elicited values. Experts were asked to be near a computer, but this was not feasible in all cases. Although it is important to obtain live feedback when fitting distributions, the results observed in our study were consistent with published estimates, where they were available for comparison. Calibration is an important element when using expert judgement. It allows for experts with different degrees of experience to be recognised when pooling the data. In this study, we chose to assume all experts had equal weight in their judgements and did not calibrate the estimates. To calibrate the estimates, it is necessary to ask experts a ‘seed’ or ‘test’ question and then adjust the weights assigned to their estimates for each parameter based on this response. There is no agreed standard about how to select appropriate seed questions and the resulting seed-derived weights have often proven to be problematic in the elicitation literature [24, 51]. Therefore, the simplistic approach of assuming equal weights for each expert was deemed to be the best strategy.

## Conclusion

This study used expert judgment to produce point estimates and measures of uncertainty for key parameters needed to describe current pathways to diagnose VFFs and facilitate the subsequent early economic evaluation of potential new approaches to improve health outcomes for people with VFFs. The results of this study support the belief that the current levels of VFF reporting and subsequent referral to appropriate care are low. Failure to detect VFFs results in high morbidity and mortality. These results can be used as a starting point to understand the potential healthcare costs and health consequences of using AI-based systems to enable the timely diagnosis of VFFs and timely initiation of bone health management programmes.

## Supplementary Information

Below is the link to the electronic supplementary material.

Supplementary file1 (DOCX 162 KB)

## Data Availability

The pre-specified protocol used to collect the data for this study is made available in the supplementary appendices. The data generated by the elicitation exercise remain the intellectual property of The University of Manchester.
