# Supplementary material for: Understanding current UK practice for the incidental identification of vertebral fragility fractures from CT scans: an expert elicitation study
Source: Aging Clin Exp Res. 2022 Apr 18;34(8):1909–18. doi: 10.1007/s40520-022-02124-w (PMC9283144; doi:10.1007/s40520-022-02124-w)
Supplement: Supplementary file 1 — Supplementary file1 (DOCX 162 KB) [file 40520_2022_2124_MOESM1_ESM.docx]

**List of supplementary appendices for:** Understanding current UK practice for the incidental identification of vertebral fragility fractures from CT scans: an expert elicitation study by Garima Dalal, Paul A. Bromiley, Eleni P. Kariki, Shawn Luetchens, Timothy F. Cootes, Katherine Payne. Osteoporosis International.

*Corresponding author*: Katherine Payne [Katherine.payne@manchester.ac.uk](mailto:Katherine.payne@manchester.ac.uk)

Online Resource 1: Reporting criteria for an expert elicitation study

Online Resource 2: Values to be estimated using expert elicitation

Online Resource 3: Study protocol

Online Resource 4: Example score sheet

Online Resource 5: Individual expert distributions of the values elicited for each parameter

Online Resource 6: Aggregated distributions of the values elicited for each parameter

***Online Resource 1****: Reporting criteria^a^ for an expert elicitation study*

| **Criterion** | **Description** | **Note** | **Reported on page** |
| --- | --- | --- | --- |
| Research rationale | The need for using an expert elicitation exercise should be described**.** | This should ideally include some reference to the design and conduct of systematic reviews to identify key input parameters for the decision analytic model and a statement confirming that these reviews did not identify data relevant for the model-based economic analysis as specified**.** | Page 7 |
| Research problem | All uncertain quantities (model input parameters) that will be elicited should be described**.** | In some instances, there may be a substantial number of uncertain quantities required, and a degree of 'pre-selection' will have occurred to identify a relevant sub-set. Clear justification for model parameters identified as key for the decision problem needs to be provided. | Page 7 and Online Resource 2 |
| Measurement type of uncertain quantities | The rationale for the measure type of each uncertain quantity elicited should be described**.** | The measurement type of uncertain quantities can be (but not limited to): scalar quantities (i.e. numbers); proportions (e.g. probabilities); ratios (e.g. odds, hazard); risk (e.g. relative); rate (e.g. mortality), etc. Some measures are easier to understand and elicit than others thus it is important to fully justify the selection of any measurement type. | Pages 6- 7 |
| Definition of an expert | The nature of the expert population should be described to clearly state what topic of expertise they represent and why**.** | It is unlikely that a single expert will be sufficient and it is generally necessary to elicit judgement from a group of experts that were selected to represent the views of a larger population**.** | Pages 7-8 |
| Number of experts | The selection criteria and final number of experts recruited to provide expert judgement should be reported. | Selection criteria need to be described in detail. There should be clear and specific pre-defined criteria used to identify how experts were selected and if/how their elicited quantities were used. | Page 9 |
| Preparation | There should be clear reference made to a protocol that describes the design and conduct of the elicitation exercise. | None. | Mentioned on page 7 (Online Resource 3) |
| Piloting | It should be clearly reported if the elicitation exercise process was piloted and a summary of any modifications made. | The selection and number of experts used in the piloting process should be reported. Key aspects that may have required modification include: selection of experts; measure type and number of uncertain quantities to be elicited; training exercise; framing of the elicitation question; method of aggregation. | Page 8 |
| Data collection | The approach to collect the data should be reported. | Data can be collected from individual experts or a group/s of experts. Collecting data from individual experts means that a mathematical aggregation process may need to be used. Collecting data from a group/s of experts means that behavioural aggregation methods may be used. | Pages 8-9 |
| Administration | The mode of administering the elicitation exercise should be reported. | Elicitation exercises can be conducted face-to-face or via the telephone and/or computer. In a limited number of situations it may be feasible to collect the data using a self-administered online or postal survey but this is unlikely to be successful in most instances. Both face-to-face and telephone data collection is likely to be supported by using a computer. | Page 8 |
| Training | The use of training materials should be reported and made available. | This may include background training materials sent to the experts and/or training in the use of probabilities and nature of distributions. This document need to provide explanation of efforts made to prevent influencing experts' knowledge and judgement. In practice, this recommendation will require a copy of the elicitation exercise to be included, which is likely to be presented as electronic supplementary material | Pages 8-9 |
| The exercise | The number and framing of questions used in the exercise should be reported and made available. | This will require a copy of the elicitation exercise to be included, which is likely to be presented as electronic supplementary material. | Page 8 |
| Data aggregation | The type of aggregation method (mathematical or behavioural) should be reported together with a description of the method or process used to aggregate the data. | Mathematical aggregation (relevant when data were collected from multiple individual experts) can be conducted using a range of methods, for example: Bayesian methods; opinion pooling; Cooke's method. Behavioural aggregation (relevant when data were collected from group/s of experts) can be conducted using processes such as, for example: Delphi or Nominal Group technique. | Page 9 |
| Measures of performance for data aggregation | The processes followed to estimate measures of performance (calibration/information) for data aggregation need to be fully described**.** | Calibration is the process of measuring the performance of experts by comparing their judgement with a 'seed parameter' (parameter whose true values are known or can be found within the duration of a study). Calibration scores represent the probability that any differences between expert's probabilities and observed values of 'seed parameters' might have arisen by chance. Information represents the degree to which an expert's distribution is concentrated, relative to some user-selected background measure. | Not applicable – study assumed equal weight for all experts |
| Ethical issues | The ethical issues for the expert sample and research community should be described. | The use of expert elicitation should acknowledge the issues of ethical responsibility, anonymity, reliability, and validity in an ongoing manner throughout the data collection and aggregation process. | Page 7 |
| Presentation of results | The individual, and aggregated, point estimate(s) and distribution for each uncertain quantity (quantities) should be presented. | **T**he units of measurement should be clear and attention should be paid to the style of presentation that may benefit from the use of figures rather than relying on a tabular format. | Pages 9 -10, Online Resources 5 and 6 |
| Interpretation of results | The interpretation of uncertain quantities elicited should be presented together with a description of how the results will be used in the model-based economic analysis. | This should include an explanation of how the reader should interpret the results. It should be recognised that the number and type of experts used will affect the results obtained. The interpretation of results should comment on the degree of uncertainty observed. | Pages 9-10 |

*^a^Source*: Iglesias CP, Thompson A, Rogowski WH, Payne K. Reporting Guidelines for the Use of Expert Judgement in Model-Based Economic Evaluations. Pharmacoeconomics. 2016;34(11):1161-72.

***Online Resource 2:*** *Values to be estimated using expert elicitation*

**CT**, computed tomography; **GP,** general practitioner; **DXA**, Dual-energy X-ray absorptiometry; **VFF** vertebral fragility fracture

| **Parameter** | **Description** | **Description of experts who quantified this parameter** |
| --- | --- | --- |
| Probability of VFF being correctly reported by the radiologist | The proportion of individuals in a population whose VFF was correctly reported by the radiologist reading the CT scan (radiologist sensitivity) | Imaging scientist (28 years of experience),  Consultant in orthogeriatric medicine (30 years of experience),  Consultant radiologist (19 years of experience),  CEO of Optasia Ltd. (company developing a ML-CAD system) (33 years of experience) |
| Probability of absence of VFF being correctly assessed by the radiologist | The proportion of individuals in a population correctly identified as not having a VFF by the radiologist reading the CT scan (radiologist specificity) | Imaging scientist (28 years of experience),  Consultant rheumatologist (26 years of experience),  Consultant radiologist (19 years of experience),  CEO of Optasia Ltd. (company developing a ML-CAD system) (33 years of experience) |
| Probability of being referred for management when a VFF is identified by the radiologist | The proportion of individuals in a population referred for management when a VFF is identified by the radiologist | Consultant rheumatologist (26 years of experience),  Consultant radiologist (19 years of experience),  Academic GP (37 years of experience),  Consultant in orthogeriatric medicine (30 years of experience),  Consultant endocrinologist (35 years of experience) |
| Probability of having a DXA scan after GP referral | The proportion of individuals in a population referred for a DXA scan after referral to a GP | Consultant rheumatologist (26 years of experience),  Consultant radiologist (19 years of experience),  Academic GP (37 years of experience),  Consultant endocrinologist (35 years of experience) |
| *^a^ Years of experience is defined as the time for when they qualified in their chosen discipline* | | |

***Online Resource 3:*** *Study protocol*

**Background**

It is generally accepted that the osteoporotic vertebral fragility fracture (VFF) identification rate is low across the globe. Current practice includes radiologists identifying and reporting VFFs that are incidentally visualised on medical images acquired for other clinical indications. However, there is evidence to suggest that around 70% of VFFs are missed by radiologists due to a lack of standardization of the definition of VFFs and their clinically silent nature. There are various machine-learning-based computer-aided diagnostic (ML-CAD) systems currently in development. The purpose of these complex interventions is to incidentally identify and grade VFFs on computed tomography (CT) scans where the spine is visible. Some ML-CAD systems are able to provide a teleradiology service where it is also possible to refer the patient with an identified VFF to their general practitioner (GP), or the local Fracture Liaison Service (FLS) if one exists.

Before complex interventions such as ML-CAD systems can be introduced into current practice, it is important to ensure that they are a cost-effective use of healthcare resources. This can be achieved by conducting an economic evaluation. However, before an economic evaluation can be undertaken, it is important to ensure that current practice can be clearly described using the available evidence. Little data exists on the current rates of early identification of VFFs by radiologists in the UK, meaning that economic models evaluating the cost-effectiveness of ML-CAD systems may not be able to demonstrate the potential gain in health benefits without considerable uncertainty associated with them. Therefore, this expert elicitation aims to quantify key parameters for informing the current practice arm of a model-based cost-effectiveness analysis.

**Identification and recruitment of experts**

Potential study participants will be identified through existing links and collaborations within the research team along with publicly available websites. The group of experts will comprise researchers involved in the identification of VFFs on CT scans as well as healthcare professionals. The experts will not be offered any financial incentives.

**Elicitation process**

All experts agreeing to take part will be invited via email to participate in face-to-face or semi-structured telephone interviews on an individual basis. The interviews will be approximately 1 hour long and will be guided by a set of open and closed questions. A laptop will be used to facilitate the exercise by showing the elicitation exercise sheet (blank box plot) to the expert during face-to-face interviews. Experts participating via a telephone interview will be asked to have the elicitation sheet open in front of them on a computer screen if possible. Telephone interviews will be arranged for individuals outside the university due to time and budget constraints.

Prior to the interview, a copy of the elicitation exercise sheet and details about the project will be shared with the experts. The elicitation exercise will involve first explaining the task and the relevant terminology to the expert, followed by an example exercise unrelated to the research problem to aid understanding of the exercise. Experts will then be asked to consider a population of individuals aged 70 years who have been referred for a CT scan in the NHS and provide values for the specified parameters. The questions will be asked in the format of, for example, “imagine a cohort of 100 patients with a VFF, how many do you think would have their VFFs being correctly reported by the radiologist?”

The expert elicitation exercise will use the quartile method where each expert will be asked to specify the highest and lowest plausible values first (i.e. a plausible range), followed by the median and upper and lower quartile values.

**Data aggregation**

A beta distribution will be fitted to the elicited values from each expert for each quantity of interest using the SHELF package in R. These individual distributions will then be linearly pooled using WinBUGS to generate an aggregate distribution representing the joint group uncertainty across all experts. The WinBUGS model will run using two Monte Carlo Markov chains with an initial ‘burn-in’ which will be determined using the Gelman-Rubin convergence diagnostic and a visual assessment of trace plots of the sample values. A further number of iterations will run after the ‘burn-in’ based on the accuracy of the posterior aggregated estimates which will be assessed by checking that the estimated Monte Carlo errors of the pooled distributions are less than 5% of the sample standard deviations. The summary measures obtained from the pooled distributions (mean and standard deviation) will be used to calculate the alpha and beta values of the beta distribution for each parameter using the method of moments.

***Online Resource 4:*** *Example of a score sheet*

**
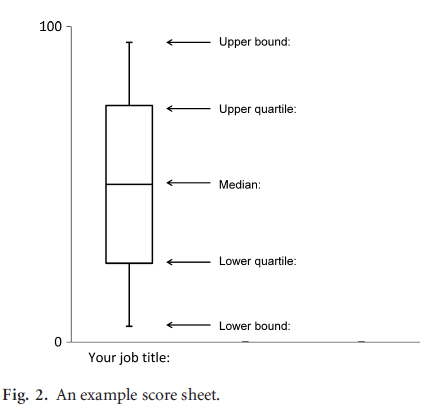
**

***Online Resource 5:*** *Individual expert distributions of the values elicited for each parameter*

**Figure S1** Probability of vertebral fragility fracture (VFF) being correctly reported by the radiologist

**Figure S2**: Probability of the absence of vertebral fragility fracture (VFF) being correctly assessed by the radiologist

**Figure S3** Probability of being referred for management when a vertebral fragility fracture (VFF) is identified by the radiologist

**Figure S4** Probability of having a dual-energy X-ray absorptiometry (DXA) scan after general practitioner (GP) referral

***Online Resource 6:*** *Aggregated distributions of the values elicited for each parameter*

This reports the observed distributions, specified using the beta distribution, for each of the four parameters:

Probability of a vertebral fragility fracture (VFF) being correctly reported by the radiologist; probability of the absence of VFF being correctly assessed by the radiologist; probability of being referred for management when a VFF is identified by the radiologist; probability of having a DXA scan after GP referral.

**Figure S6** Probability of a vertebral fragility fracture (VFF) being correctly reported by the radiologist

*Beta (α = 0.84, β = 2.48) where the two parameters (α, β) define the observed shape of the distribution.*

**Figure S7** Probability of the absence of vertebral fragility fracture (VFF) being correctly assessed by the radiologist

*Beta (α = 7.59, β = 0.93) where the two parameters (α, β) define the observed shape of the distribution.*

**Figure S8** Probability of being referred for management when a vertebral fragility fracture (VFF) is identified by the radiologist

*Beta (α = 1.15, β = 6.73) where the two parameters (α, β) define the observed shape of the distribution.*

**Figure S9** Probability of having a dual-energy X-ray absorptiometry (DXA) scan after general practitioner (GP) referral

*Beta (α = 1.21, β = 0.61) where the two parameters (α, β) define the observed shape of the distribution.*
